# Synergistic consequences of early-life social isolation and chronic stress impact coping and neural mechanisms underlying male prairie vole susceptibility and resilience

**DOI:** 10.3389/fnbeh.2022.931549

**Published:** 2022-07-25

**Authors:** Lindsay L. Sailer, Pooja P. Patel, Ashley H. Park, Joanna Moon, Amit Hanadari-Levy, Alexander G. Ophir

**Affiliations:** Department of Psychology, Cornell University, Ithaca, NY, United States

**Keywords:** social isolation, chronic social defeat stress (CSDS), comfort-seeking, social support, stress resilience, lateral septum, gene concordance

## Abstract

Chronic stress can be challenging, lead to maladaptive coping strategies, and cause negative mental and physical health outcomes. Early-life adversity exposes developing young to physical or psychological experiences that risks surpassing their capacity to effectively cope, thereby impacting their lifetime physical and mental wellbeing. Sensitivity to stressful events, like social isolation, has the potential to magnify stress-coping. Chronic stress through social defeat is an established paradigm that models adverse early-life experiences and can trigger enduring alterations in behavioral and neural phenotypes. To assess the degree to which stress resilience and sensitivity stemming from early-life chronic stress impact sociability, we exposed male prairie voles to chronic social defeat stress (CSDS) during adolescence. We simultaneously exposed subjects to either social isolation (CSDS+Isol) or group housing (CSDS+Soc) during this crucial time of development. On PND41, all subjects underwent a social approach test to examine the immediate impact of isolation, CSDS, or their combined effects on sociability. Unlike the CSDS+Isol group which primarily displayed social avoidance, the CSDS+Soc group was split by individuals exhibiting susceptible or resilient stress phenotypes. Notably, the Control+Soc and CSDS+Soc animals and their cage-mates significantly gained body weight between PND31 and PND40, whereas the Control+Isol and CSDS+Isol animals did not. These results suggest that the effects of early-life stress may be mitigated by having access to social support. Vasopressin, oxytocin, and opioids and their receptors (*avpr1a, oxtr, oprk1, oprm1*, and *oprd1*) are known to modulate social and stress-coping behaviors in the lateral septum (LS). Therefore, we did an mRNA expression analysis with RT-qPCR of the *avpr1a, oxtr, oprk1, oprm1*, and *oprd1* genes to show that isolation and CSDS, or their collective influence, can potentially differentially bias sensitivity of the LS to early-life stressors. Collectively, our study supports the impact and dimensionality of early-life adversity because the type (isolation vs. CSDS), duration (acute vs. chronic), and combination (isolation + CSDS) of stressors can dynamically alter behavioral and neural outcomes.

## Introduction

Prolonged social isolation or negative social relationships can adversely affect physical and emotional health (Johnson et al., 2000). Social isolation has been linked to cognitive impairment, reduced immunity, increased risk of cardiovascular disease, increased risk of depression, suicidal thoughts, and risk of early mortality (Cacioppo and Cacioppo, 2014). The effects of social isolation are compounded in individuals who lack a strong system of social support. Indeed, social isolation and low levels of social support are linked with higher levels of stress, depression, posttraumatic stress disorder, and increased morbidity and mortality in a host of trauma-related psychopathologies (Southwick et al., 2005). Positive social support, on the other hand, is essential for maintaining physical and psychological health by preventing the development of or decreasing the consequences of trauma-related mental disorders, reducing morbidity and mortality, and serving as a buffering mechanism to enhance resilience (Southwick et al., 2005; Ozbay et al., 2007). Adolescence is a sensitive period of physical and mental development that takes place from puberty to adulthood, or at a time of life when individuals can achieve self-sufficiency (Blakemore and Mills, 2014). This period of formative biological and social transition is characterized by increased risk-taking, sensation-seeking, moodiness, and drug use (Lipari and Jean-Francois, 2013; Fuhrmann et al., 2015; Kilford et al., 2016). Adolescence is associated with the onset of psychiatric disorders, such as depression, anxiety, impulse-control disorders, and phobias (Kessler et al., 2005, 2007). Furthermore, stress exposure during adolescence may be a contributing factor in triggering psychiatric disorders (Andersen and Teicher, 2008).

Like early human development, interactions with age-matched conspecifics are critical for adolescent development in rodents. Social play (e.g., playfighting) is essential for the maturation of the brain and behavior and begins during pre-adolescence and peaks during mid-adolescence in rodents (Panksepp, 1981; Pellis and Pellis, 2017). Depriving adolescent rats and mice of social play causes long-term effects on adult behavioral outcomes, including susceptibility to drug addiction (Zakharova et al., 2009; Whitaker et al., 2013), increased anxiety-like and depression-like behaviors (Einon and Morgan, 1977; Arakawa, 2005, 2007; Amiri et al., 2015), and social interaction and social approach deficits (Van Den Berg et al., 1999; Lukkes et al., 2009a,b). The enhanced anxiety states in rats isolated during adolescence cannot be reversed if they are re-socialized with other isolated rats (Einon and Morgan, 1977; Arakawa, 2005, 2007). Notably, rats isolated during adolescence are protected from developing heightened adult defensive aggression if they are allowed 1-h daily social play sessions with isolated conspecifics (Potegal and Einon, 1989). Conversely, social rejection or co-housing rats with non-playful adults or atypical conspecifics throughout adolescence induces enduring impairments in social interaction, social memory, pain sensitivity, and neural development (Einon et al., 1978; Bell et al., 2010; Schneider et al., 2016). Thus, the deleterious effects of social isolation during adolescent development may, in part, emerge from an absence of reciprocal social exchanges or inadequate social dynamics.

Positive social relationships during adolescent development are fundamental to emotional and behavioral adjustment, cognitive function, and perceptions of safety (Richards and Wadsworth, 2004; Mensah et al., 2013; Smith and Pollak, 2021). Indeed, individuals experiencing chronically stressful events during adolescence are at greater risk of developing long-term and irreversible negative impacts on metabolism, brain development, reproductive function, immune function, cardiovascular function, and behavior compared to individuals who do not experience high levels of stress (Charmandari et al., 2003; Pervanidou and Chrousos, 2007, 2012). Notably, whereas some individuals who have experienced chronic stress develop signs of depression after traumatic experiences, others continue to persevere in the face of adversity and show resilient traits such as cognitive flexibility and optimism (Charney, 2004; Yehuda et al., 2006). In some cases, social support grants protective and buffering effects against stress, thereby enhancing mental and physical health and fostering effective coping strategies (Southwick et al., 2005, 2016). Therefore, social support could prove to be a key factor in promoting stress resilience. However, the behavioral and physiological responses to chronic stress, such as social isolation or physical trauma, are shaped by environmental and genetic factors that interact in a poorly understood manner (Nestler et al., 2002).

Animal models of stress have called attention to the consequences of social stressors. The Chronic Social Defeat Stress (CSDS) paradigm is an ecologically validated assessment of using repeated exposure to social defeat to generate persistent emotional and psychological stress without habituation (Golden et al., 2011). Social defeat is defined as an individual losing a social confrontation in the home territory of an older, aggressive, and dominant conspecific (Golden et al., 2011; Hollis and Kabbaj, 2014). In rodents, CSDS produces a range of phenotypes that parallel interindividual variability observed in humans who suffer from chronic emotional or physical stress. Socially defeated rodents are characterized as being susceptible or resilient to chronic stress based on behavioral responses evaluated by a novelty-based social approach test following CSDS. Accordingly, the CSDS paradigm provides a valuable opportunity to investigate the neural and molecular basis of susceptibility and resilience to chronic stress (Alves-dos-Santos et al., 2020).

The majority of work investigating CSDS has shown that repeated exposures to social defeat stress in mice causes a robust depression-like phenotype marked by increased social withdrawal, increased immobility in the forced swim test, reduced sucrose preference (a measure of anhedonia), physiological dysregulation, and decreases in reward sensitivity (Golden et al., 2011; Iñiguez et al., 2014; Sial et al., 2021). Much of this work has been extremely beneficial to understanding how early experiences can predispose animals to cope with stressful events experienced in later life. Studies involving numerous central and peripheral body systems, such as brain circuitry, gut microbiota, immune system, and blood-brain barrier, have shown that resilience to stress is a multifaceted process that affects humans and mice alike and varies by sex across the lifespan (Cathomas et al., 2019; Hodes and Epperson, 2019).

Much of the aforementioned work is limited in translational relevance because mice might be generally social, but do not demonstrate a system of persistent selective social bonds. Indeed, social bonds have been shown to help buffer stress by suppressing cortisol release, reducing blood pressure reactivity, and attenuating pain sensitivity (Heinrichs et al., 2003; Roberts et al., 2015; Howard et al., 2017). For this reason, assessing questions focused on understanding the interaction of chronic stress in the development and later stress resiliency/susceptibility would be enhanced by studies involving animals with a strong capacity to form social bonds. Prairie voles offer such an opportunity and are widely recognized for their ability to form selective bonds and demonstrate selective attachment (Getz et al., 1981; Carter and Getz, 1993). The importance of social bonding between peers and mates in prairie voles is underscored by isolation-related behavioral and physiological impairments. In prairie voles, separation from peers is associated with causing anxiety-like and depression-like behaviors, such as decreased exploratory behavior in the elevated plus-maze, a progressive decline in sucrose intake, and increased immobility in the forced swim test (Stowe et al., 2005; Grippo et al., 2007, 2008). Peer separation is also associated with changes in neuroendocrine signaling and cardiac function (Grippo et al., 2007, 2011). Separation from mating partners leads to increased passive stress-coping strategies and increases heart rate in prairie voles (Bosch et al., 2009; McNeal et al., 2014). Like mice, social defeat stress causes social avoidance in prairie voles (Tickerhoof et al., 2020; Hale et al., 2021).

Importantly, early-life stress and social support can impact brain physiology and development. Although numerous aspects of neural modulation are sensitive to early-life stress, vasopressin (VP) and oxytocin (OT) are of particular interest because they modulate activity in various brain regions critical for a variety of social behaviors (Goodson and Thompson, 2010; Prounis and Ophir, 2020). Of the many nodes of the social brain, the lateral septum (LS) stands out as potentially central for governing context-specific motivational states and behavioral responses appropriate to environmental stimuli (Sheehan et al., 2004; Luo et al., 2011; Sartor and Aston-Jones, 2012; Prounis and Ophir, 2020). Indeed, the LS is important for social recognition, social motivation, formation of partner preferences, selective aggression, and parental care in a variety of species (Liu et al., 2001; Ophir et al., 2009; Curley et al., 2012; O'Connell et al., 2012) and is sensitive to changes in both VP and OT systems after exposure to early-life stress. For example, male, but not female, prairie vole offspring raised under conditions in which parents prioritize caring for themselves over their offspring shows social deficits and exhibits upregulation of the vasopressin receptor (*avpr1a)*, but not oxytocin receptor (*oxtr*), mRNA in the LS (Kelly et al., 2020). Similarly, prairie voles which were raised by mothers only, and then socially isolated after weaning demonstrated impairments in socio-spatial memory, greater oxytocin receptor density in the LS, and a neural phenotype across areas of the brain closely associated with contextual memory that replicated a neural phenotype that is associated with remaining unpaired in naturalistic outdoor field conditions (Prounis et al., 2015). CSDS evokes a variety of responses in the VP and OT systems (Kompier et al., 2019). In the mice, oxytocin receptor-expressing neurons co-express significantly more c-Fos, an immediate early gene marker of neural activity, after social defeat stress (Nasanbuyan et al., 2018). Studies in several socially monogamous species, such as California mice, mandarin voles, and prairie voles, have shown that social defeat alters oxytocin receptor levels in the brain regions associated with social behaviors (Duque-Wilckens et al., 2018; Wang et al., 2018; Hou et al., 2020; Hale et al., 2021). Notably, recent studies have shown that early-life stress impacts the endogenous opioid system, including the opioid receptors *oprk1, oprm1*, and *oprd1* (Chang et al., 2019).

The goal of our study was to characterize the developmental impact of social isolation, CSDS, or the collective impact of both stressors on sociability and neural correlates in juvenile male prairie voles. We modified the standardized CSDS protocol (Golden et al., 2011) to permit half of the male subjects to experience either social isolation or social support during CSDS. Vasopressin, oxytocin, and opioid receptor mRNA expression levels were measured at adolescent time points to assess the immediate changes in the development of the vasopressin, oxytocin, and opioid systems by early-life stress. Because social bonding and social support are related to better stress-coping mechanisms and stress alleviation, we predicted that affiliative social support would buffer the impact of CSDS on social approach behavior, whereas social isolation would heighten social deficits induced by CSDS. Owing to previous work implicating the vasopressin, oxytocin, and opioid systems in stress, social isolation, and depressive behaviors, we also predicted that social isolation and CSDS would alter mRNA expression. Taken together, this study aimed to understand how social isolation, chronic social defeat stress, and their interaction can alter sociability, and elucidate signaling pathways with the potential to alter neural responses to discreet or compounded effects of early-life stress.

## Materials and Methods

### Animals

**Figure 1:**
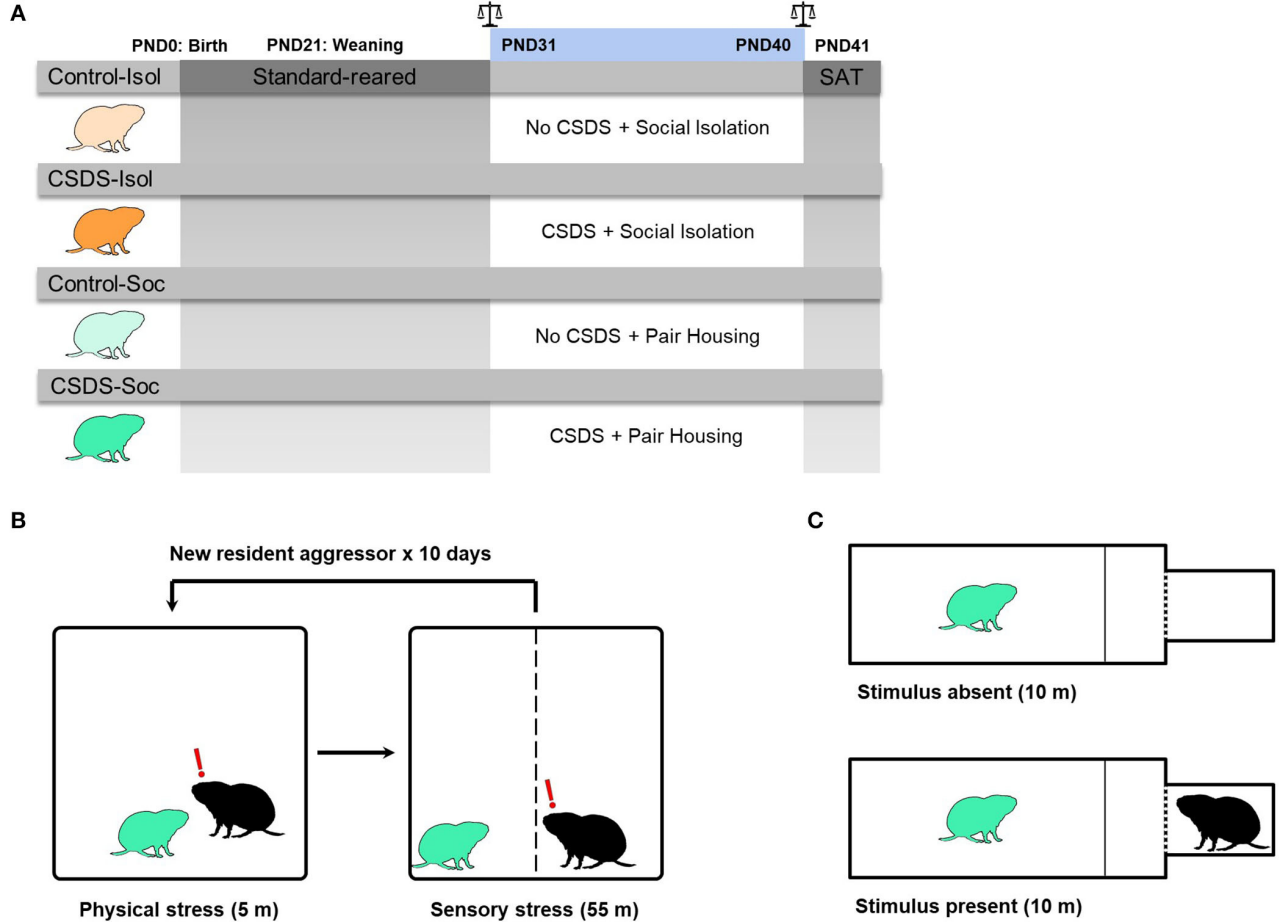
Experimental design. **(A)** Graphical representation of the experimental timeline. Male prairie voles were standard-reared between PND0-30. In adolescence (PND31), voles from each group experienced either control conditions (Control: light orange or light green voles) or 10 days of chronic social defeat stress (CSDS: dark orange or dark green voles). After the first social defeat session, half of the voles remained in social isolation (Control+Isol and CSDS+Isol) and the other half remained pair housed with a same-sex cage-mate (Control+Soc and CSDS+Soc). Scale signs indicate that body weights of subjects and cage-mates were measured before and after CSDS, PND31 and PND40, respectively. Subjects and their cage-mates were assessed for social avoidance in the social approach test (SAT), before they were immediately sacrificed for brain tissue harvesting and gene expression analysis. **(B)** Diagram representing the CSDS procedure. The CSDS+Isol (dark orange) and CSDS+Soc (dark green) groups experienced CSDS, while the Control+Isol (light orange) and Control+Soc groups (light green) were placed into an empty and clean cage. **(C)** Diagram representing the social approach test (SAT) when the stimulus is absent (i.e., habituation phase) and when the stimulus is present (*i.e.*, testing phase). All groups were assessed in the SAT.

The prairie voles used in this study were obtained from our laboratory breeding colony, from pairs that were offspring of wild-caught animals in Champaign County, Illinois, United States. The subject males were weaned from breeding pairs on postnatal day (PND) 21 and pair-housed with a same-sex littermate until PND30 (Figure 1A). The subjects were randomly selected to remain pair-housed with their same-sex littermate or to endure social isolation after each social defeat session and until the social approach test (PND31-41), thus yielding 4 groups (n = 10 males/group): 1) a stress-naïve and socially isolated group (Control+Isol), 2) a CSDS and socially isolated group (CSDS+Isol), 3) a stress-naïve and socially housed group (Control+Soc), and 4) a CSDS and socially housed group (CSDS+Soc). The subjects were assigned to different groups when ≥3 males were born to the same litter to circumvent litter effects. All animals were housed in standard polypropylene cages (46.5 × 25 × 15.5 cm) containing sani-chips bedding and provided nesting material, and kept on a 14:10 light:dark cycle, with lights on at 0800 h. Rodent chow (Laboratory Rodent Diet 5001, LabDiet, St. Louis, MO, United States) and water were provided *ad libitum*. Ambient temperature was maintained at 20 ± 2°C. All procedures were approved by the Animal Care and Use Committee of Cornell University (2013-0102) and were as per the guidelines set forth by the National Institutes of Health.

### Body weight measurement

Body weight for male subjects and their cage-mates was recorded before the first (PND31) and after the last (PND40) social defeat sessions (Figure 1A). Weight gain was calculated as: body weight (g) at PND40 – body weight (g) at PND31.

### Chronic social defeat stress

The CSDS paradigm was modeled after an established CSDS protocol for mice (Golden et al., 2011), with some modifications relevant to voles. In mice, a social defeat session typically involves a 5-min physical stress phase (the social confrontation) followed by a 24-h sensory stress phase that allows prolonged auditory, visual, and olfactory interactions between the subject and resident. However, some studies have demonstrated that chronic physical defeat alone is sufficient to induce behavioral changes (Dietz et al., 2008, 2011). Here, we modified the CSDS protocol to permit the defeated prairie voles to return to their home cages after truncated social defeat sessions that involved physical stress limited to 3 attacks and sensory stress limited to 55 min (Figure 1B). This modified protocol avoided the possibility for pair bonding-induced aggression to decline in the residents and allowed us to investigate how social support impacts sociability and neural responses to social isolation, CSDS, and the combined effects of both stressors in juveniles.

Pair bonded and actively breeding male prairie voles (90–120 days old) were screened for aggressiveness. Those that attacked a novel sex-naïve adolescent male intruder ≥3 times within a 5 min period, on three consecutive screening sessions, were chosen as the resident aggressors to defeat subjects in this study. A total of 10 out of 16 pair bonded males were chosen as resident aggressors. Adolescent subject males were introduced into the home cage of a novel resident aggressor and allowed to be attacked by the resident up to 3 times within a 5 min period. After the first 3 attacks or 5 min of physical stress, a perforated plexiglass divider was inserted to separate the subject from the resident aggressor to permit continued sensory stress for 55 min. The 60 min social defeat session of physical (5 min) and sensory (55 min) stress was repeated once daily for 10 consecutive days with a novel resident aggressor for subjects (PND31-40) in the CSDS+Isol and CSDS+Soc groups. The Control+Isol and Control+Soc subjects were placed into an empty and clean cage for 60 min daily (PND31-40), with the plexiglass divider inserted for the last 55 min, and were not video recorded.

### Social isolation

Immediately after the first social defeat session, subjects from the Control+Isol and CSDS+Isol groups were socially isolated in polypropylene cages (29 × 18 × 13 cm) containing sani-chips bedding and nesting material. In contrast, subjects from the Control+Soc and CSDS+Soc groups were reunited with their cage-mate immediately after each social defeat session. The Control+Isol and CSDS+Isol subjects remained in social isolation for 23 h between each social defeat session and the social approach test. Socially isolated subjects were housed in the same colony space as the Control+Soc and CSDS+Soc groups.

### Assessment of comfort-seeking and consoling behaviors

Affiliative behaviors, such as huddling, form and reinforce social bonds between animals and can help to comfort individuals exposed to stressful events. We recorded and scored the first 10 min following the reunion of Control+Soc and CSDS+Soc subjects with their cage-mates after the first (PND31) and last (PND40) social defeat sessions to evaluate the effects of acute and chronic social defeat stress on comfort-seeking behaviors. We quantified the duration of huddling (seconds) when either the subject or cage-mate initiated physical contact with the other to differentiate between subject comfort-seeking behaviors and cage-mate-directed consoling behaviors. The Control+Isol and CSDS+Isol subjects were returned to social isolation after each social defeat session and were not video recorded.

### Social approach test

The animals were introduced and habituated to the social approach test apparatus (20 × 40 × 28 cm) for 10 min with the stimulus absent. After habituation, a doorway separating the testing chamber from a stimulus presentation box (10.06 cm^3^) containing a novel pair bonded male was unblocked, exposing the subject to the stimulus for 10 min (Figure 1B). The stimulus chamber was separated from the testing chamber with a perforated wall allowing for visual, auditory, and olfactory contact between the subject and stimulus. The habituation and testing sessions were video recorded and the latency to approach the stimulus, social interaction ratio, distance moved (cm), and velocity moved (cm/s) were quantified. The latency to approach the stimulus was quantified as the difference in time from the start of the test until the nose of the subject was within 3 cm of the stimulus chamber. The social interaction (SI) ratio was quantified as the time spent in the social zone with the stimulus present divided by the time spent in the social zone with the stimulus absent. Subjects with an SI ratio >1.1 are defined as “social,” whereas subjects with an SI ratio < 0.9 are defined as “socially avoidant” (Golden et al., 2011; Peña et al., 2019).

### Tissue processing and RNA extractions

The male subjects were euthanized following the recommended ethical and regulatory guidelines by rapid decapitation on PND41 immediately after the social approach test. The brains were immediately extracted and frozen on powdered dry ice before being stored at −80°C. We selected coronal sections (200 μm thick) anatomically matching Plates 14–34 from Paxinos and Watson's rat brain atlas (Paxinos and Charles Watson, 2007). Tissue punches (1-mm diameter) were collected bilaterally from the lateral septum (LS) region for each subject, and stored at−80°C until further processing for total RNA extraction using TRI-reagent according to the manufacturer's protocol (Molecular Research Center) and as previously described (Wang et al., 2013; Sailer et al., 2019).

### Reverse transcription and semi-quantitative real-time PCR

Total RNA (500 ng) was reverse-transcribed with the LunaScript^TM^ RT SuperMix Kit (New England Biolabs, E2010) to examine the mRNA expression for *avpr1a, oxtr, oprk1, oprm1*, and *oprd1* RT-qPCR in triplicates (see Supplementary Table 1 for primer sequences) for each subject. Primer specificity was verified by the melt curve analysis. For each primer pair, amplified cDNA was normalized to nicotinamide adenine dinucleotide dehydrogenase (NADH) (Wang et al., 2013; Sailer et al., 2019). All data were included in the analyses unless statistically defined as an outlier (>2 standard deviations from the mean).

### Statistical analyses

**Figure 2:**
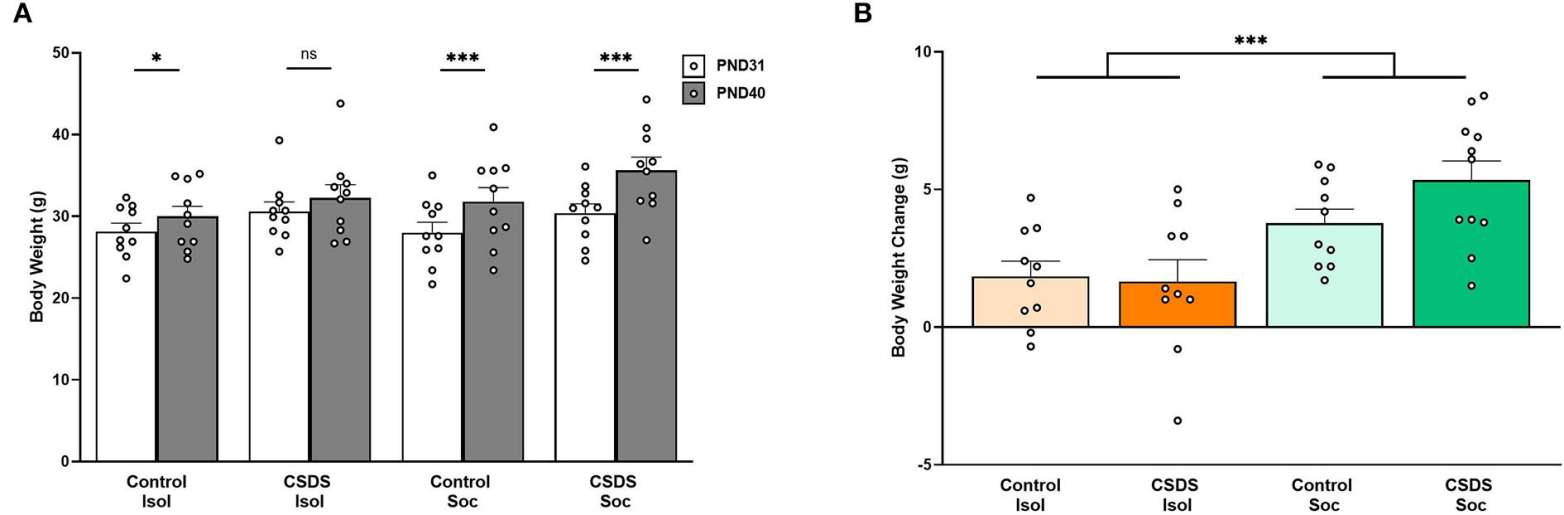
Effects of social isolation and CSDS on body weight and body weight gain. **(A)** Body weight measured before the first social defeat session on PND31 and after the last social defeat session on PND41. **(B)** Body weight gain (body weight on PND40 – body weight on PND31). Color scheme follows Figure 1 where orange = isolated; green = group-housed; dark colors = chronic social defeat; light colors = control (no chronic social defeat). Data are presented as mean ± SEM and dots represent individual data. *p < 0.05; ***p < 0.01.

**Figure 3:**
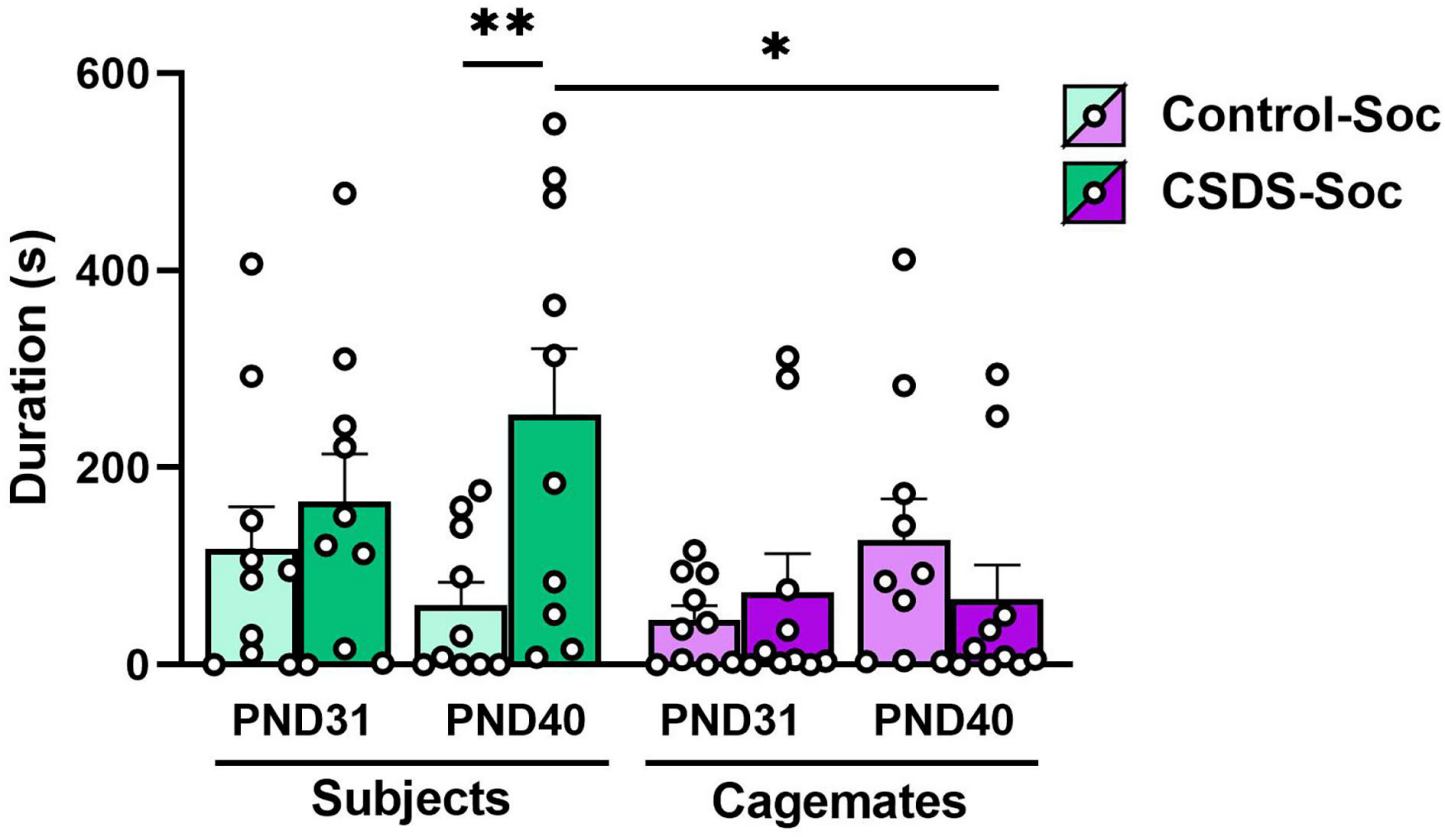
Effects of acute and chronic social defeat stress on comfort-seeking and consoling behaviors. Duration of comfort-seeking behavior by the Control+Soc (light green bars) or CSDS+Soc (dark green bars) subjects or duration of consoling behavior by the subjects' corresponding cage-mates (light purple bars for Control+Soc; dark purple bars for CSDS+Soc) after the first social defeat session (PND31) and last social defeat session (PND40). Data are presented as mean ± SEM and dots represent individual data. *p < 0.05; **p < 0.01.

**Figure 4:**
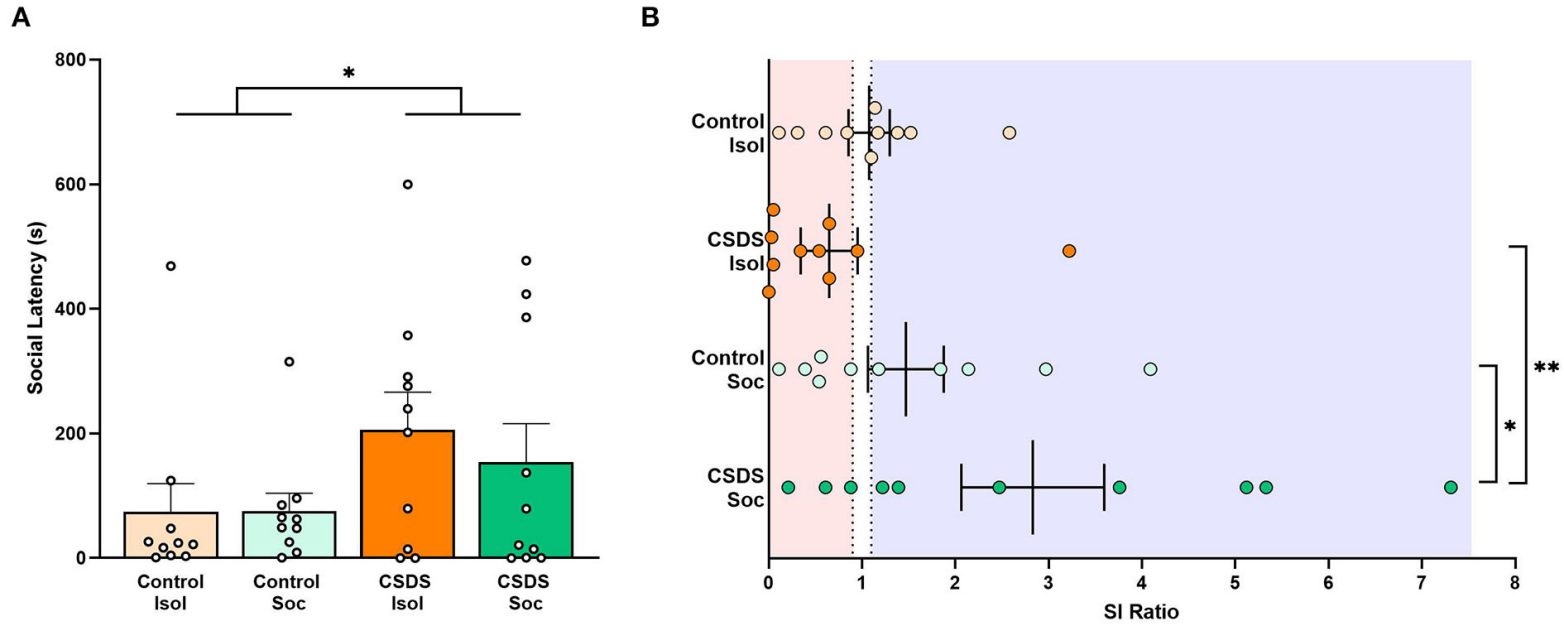
Effects of social isolation and CSDS on social approach. **(A)** Latency (s) to approach the stimulus. Data are presented as mean ± SEM and dots represent individual data. **(B)** Social interaction (SI) ratios were calculated for all subjects (= the time spent in social zone with the stimulus present divided by the time spent in the social zone with the stimulus absent). Dotted lines indicate SI ratios at 0.9 and 1.1, with red transparent panel indicating a socially avoidant phenotype (SI ratios < 0.9) and blue transparent panel indicating a prosocial phenotype (SI ratios >1.1). Color scheme follows Figure 1 where orange = isolated; green = group-housed; dark colors = chronic social defeat; light colors = control (no chronic social defeat). Data presented as mean ± SEM and dots represent individual data. *p < 0.05; **p < 0.01.

**Figure 5:**
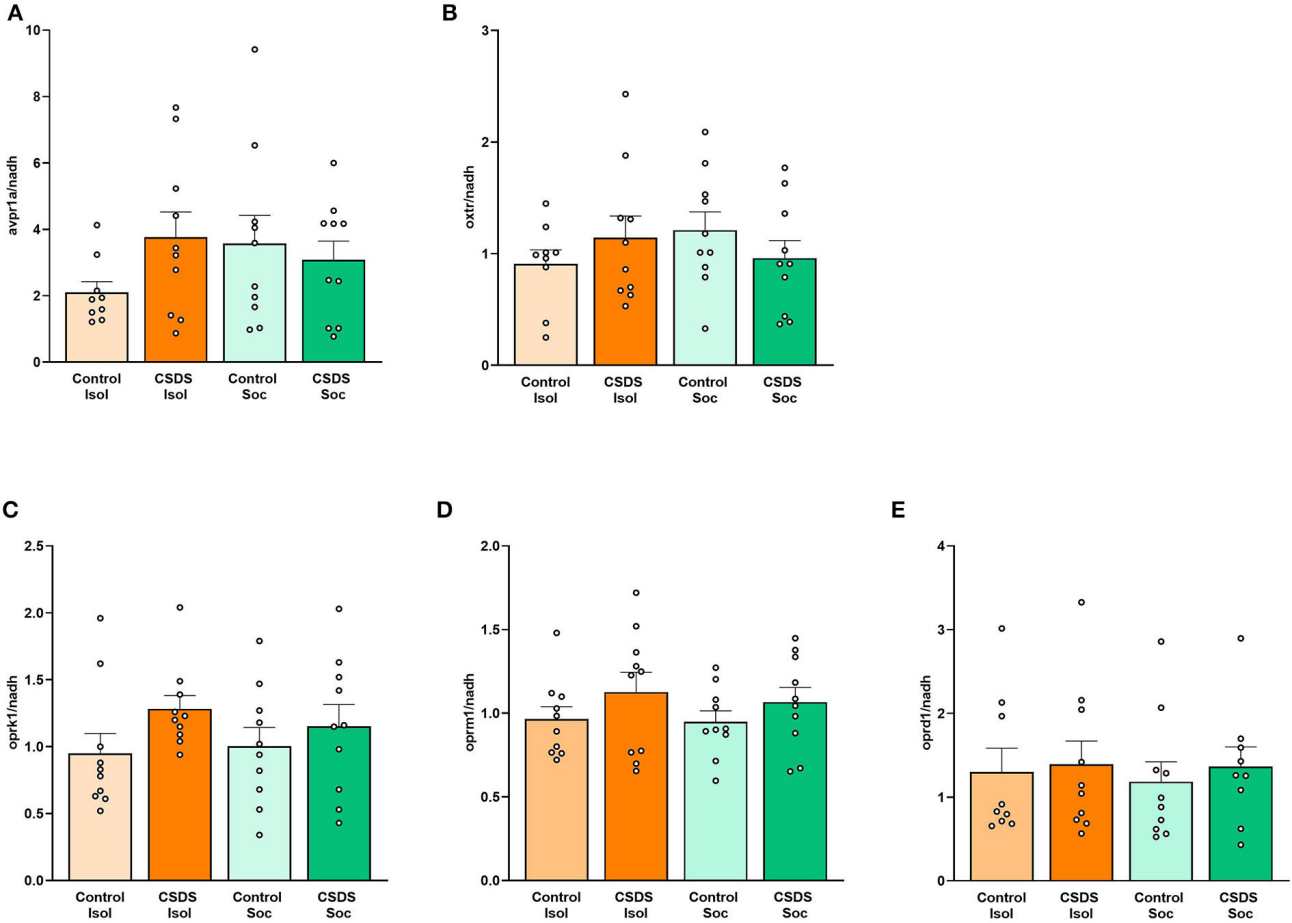
Effects of social isolation and CSDS on gene expression in the lateral septum. **(A)** *avpr1a*, **(B)** *oxtr*, **(C)** *oprk1*, **(D)** *oprm1*, and **(E)** *oprd1* mRNA levels in the LS are not significantly associated with vulnerability or resilience to isolation of social stress. Color scheme follows Figure 1 where orange = isolated; green = group-housed; dark colors = chronic social defeat; light colors = control (no chronic social defeat). Data are presented as mean ± SEM and dots represent individual data.

All behavioral procedures were manually scored by an observer blind to groups using Noldus Observer XT 11 (Noldus, Leesburg, VA, United States), Noldus Ethovision XT 13, or BORIS 7.9.7. Data were analyzed with RStudio (version 1.2.1335) using a linear mixed-model (LMM) framework with the packages lme4 (Bates et al., 2015), emmeans (Lenth et al., 2020), and lmerTest (Kuznetsova et al., 2017). Significant interactions or significant main effects (α = 0.05) were followed by Tukey's *post hoc* test (Howell, 2010). For comparisons between groups, data were tested for normality (Shapiro-Wilk) and equal variance. We performed an unpaired *t*-test (two-tailed) when data were normally distributed. If data were not normally distributed, we performed a Wilcoxon rank-sum test. Figures 2–5 were created using Prism version 9.3.1 (GraphPad Software, San Diego California United States) and all data are presented as the mean ± SEM. Body weight and weight gain were analyzed by using an LMM to compare the effects of stress (no CSDS vs. CSDS), housing (isolation and pair-housing), and postnatal day (PND31 vs. PND40) between groups. Duration of huddling between subjects and cage-mates after being reunited following the first and last social defeat sessions was analyzed to compare the effects of acute and chronic social defeat stress in the Control+Soc and CSDS+Soc subjects and cage-mates. Behaviors from the social approach test were analyzed to compare the effects of stress (no CSDS vs. CSDS) and housing (isolation and pair-housing) between groups on latency to approach the stimulus, SI ratio, distanced moved, and velocity. We performed Pearson correlations between mRNA expression within each group *p*-values were adjusted for multiple comparisons with False Discovery Rate (FDR) (Benjamini and Hochberg, 1995). The Hmisc and corrplot packages in R were used to visualize the correlograms (Wei et al., 2017; Harrell and Dupont, 2022). The pairs R function was used to visualize the multi-panel scatterplot matrices.

## Results

### Effects of social isolation and CSDS on body weight and body weight gain

The body weight was measured before the first social defeat session (PND31) and after the last social defeat session (PND40) to compare the effects of social isolation and CSDS (Figure 2A). An LMM showed that male prairie voles generally gained body weight during adolescent development (Age effect: F_(1,40)_ = 97.02, *p* = 2.98e-12), chronic social defeat impacted weight gain (Stress effect: F_(1,40)_ = 4.77, *p* = 0.04), and that being isolated or group-housed interacted with age to impact weight gain (Age x Housing effect: F_(1,40)_ = 18.92, *p* = 9.14e-5). We did not find a main effect for social or isolated housing on weight gain (Housing effect: F_(1,40)_ = 0.93, *p* = 0.34), and the other interactions did not account for weight gain differences (Age x Stress: F_(1,40)_ = 0.99, *p* = 0.33; Stress x Housing: F_(1,40)_ = 0.09, *p* = 0.77; Age x Stress x Housing: F_(1,40)_ = 1.67, *p* = 0.20). *Post hoc* comparisons showed that subjects from Control+Isol (*t*_44.4_= −2.75, estimate = −1.84, s.e. = 0.67, *p* = 0.04), Control+Soc (*t*_44.4_= −5.65, estimate = −3.78, s.e. = 0.67, *p* < 0.0001), and CSDS+Soc (*t*_44.4_= −7.82, estimate = −5.23, s.e. = 0.67, *p* < 0.0001) gained weight as they grew older, whereas the combination of early-life chronic stress (CSDS) and social isolation prevented CSDS+Isol subjects from showing significant weight gain (*t*_44.4_= −2.47, estimate = −1.65, s.e. = 0.67, *p* = 0.08). These data suggest that CSDS+Isol males might have failed to thrive.

We also assessed the weight of the cage-mates when subjects were housed socially, and found a main effect of age (LMM, Age effect: F_(1,20)_ = 13.31, *p* = 1.59e-3) with significant weight gain in both groups of cage-mates (*post hoc* Control+Soc cage-mates: *t*_22.2_= −2.81, estimate = −2.99, s.e. = 1.06, *p* = 0.01; CSDS+Soc cage-mates: *t*_22.2_= −2.09, estimate = −2.22, s.e. = 1.06, *p* = 0.049; data not shown) indicating that the siblings also significantly gained weight. The stress status of the subjects (Control+Soc and CSDS+Isol) did not impact their cage-mates' weight gain (Stress effect: F_(1,20)_ = 0.07, *p* = 0.79), and no interaction between the cage-mates' age and subjects' stress status was found (Age x Stress: F_(1,20)_ = 0.29, *p* = 0.60).

Finally, body weight gain was calculated to determine the overall effects of social isolation and CSDS (Figure 2B). A 2 (Housing) x 2 (Stress) LMM revealed a main effect of Housing (F_(1,36)_ = 17.01, *p* = 2.09e-4), indicating that social housing facilitated age-appropriate growth. *Post hoc* analyses revealed that Control+Soc subjects were significantly heavier than Control+Isol subjects (Control+Isol vs. Control+Soc *t*_36_= 2.05, estimate = 1.94, s.e. = 0.94, *p* = 0.048) and CSDS+Soc subjects were significantly heavier than CSDS+Isol subjects (CSDS+Isol vs. CSDS+Soc *t*_36_= 3.78, estimate = 3.58, s.e. = 0.94, *p* = 0.0006). The Control and CSDS isolated animals did not differ (*t*_36_= −0.20, estimate = −0.19, s.e. = 0.94, *p* = 0.84), and neither did Control and CSDS socially housed animals (*t*_36_= 1.53, estimate = 1.45, s.e. = 0.94, *p* = 0.13). The body weight gain of cage-mates of subjects assigned to the Control+Soc and CSDS+Soc subjects also did not differ (*t*_18_= −0.51, estimate = −0.77, s.e. = 1.51, *p* = 0.61).

### Effects of acute and chronic social defeat stress on comfort-seeking behaviors

It is possible that social defeat might impact how subjects interact with their cage-mates upon being reunited. This possibility raises questions like do socially defeated subjects avoid their cage-mates, and thus, prevent cage-mates from consoling them, or do they seek comfort from their cage-mates? To investigate this, we identified which animal of a socially housed pair-initiated huddling and the duration of those huddling bouts immediately after the first (PND31) and last (PND40) social defeat sessions (Figure 3). We defined huddling initiated by the cage-mate as consoling behavior and huddling initiated by the subject as comfort-seeking behavior. An LMM revealed a main effect of Animal (subject or cage-mate; Animal effect: F_(1,36)_ = 5.20, *p* = 0.03), a 2-way interaction between Animal x Stress (F_(1,36)_ = 4.77, *p* = 0.04), and a three-way interaction between Age x Animal x Stress (F_(1,36)_ = 4.55, *p* = 0.04). No other main effects or interactions were significant (Age x Animal: F_(1,36)_ = 0.15, *p* = 0.70, Age x Stress: F_(1,36)_ = 0.27, *p* = 0.60). Regardless of stress, subjects and cage-mates from both Control+Soc and CSDS+Soc engaged in similar amounts of huddling toward one another on PND31; that is, the duration of comfort-seeking by subjects was like the duration of consoling by cage-mates in both groups that day. However, after repeated exposure to social defeat (on PND40), the duration of comfort-seeking by the CSDS+Soc subjects was higher than the duration of consoling behaviors by their corresponding cage-mates (*posthoct*_70.8_ = −3.19, estimate = −187.57, s.e. = 58.8, *p* = 0.01). Notably, the duration of comfort-seeking by CSDS+Soc subjects was significantly higher than the duration of comfort-seeking by Control+Soc subjects (*posthoct*_70.8_= 3.29, estimate = 193.53, s.e. = 58.8, *p* = 0.008) on PND40. Taken together, these results demonstrate that CSDS potentiates comfort-seeking behaviors.

### Effects of social isolation and CSDS on social approach

After 10 days of social defeat, subjects were evaluated for social approach to assess the effects of social isolation and chronic social defeat stress. Similarly, the cage-mates of subjects in the social housing conditions were assessed in the social approach test to determine if stress experienced by the subject was transferred to their non-stressed cage-mates. An LMM revealed a main effect of chronic social defeat stress for the latency to approach an unfamiliar conspecific stimulus (Stress effect: F_(1,36)_ = 4.28, *p* = 0.046; Figure 4A) indicating that chronically defeated subjects were more socially averse than subjects who were not chronically stressed. No effect of housing (social vs. isolated housing) was found (Housing effect: F_(1,36)_ = 0.24, *p* = 0.62) and the interaction between housing and stress was not significant (Housing x Stress: F_(1,36)_ = 0.28, *p* = 0.60) indicating that social buffering due to social housing did not improve the social hesitancy caused by chronic social defeat stress. Notably, we found no significant differences in social approach latencies between cage-mates of the Control+Soc and CSDS+Soc subjects (unpaired *t*-test: t_18_ = 1.28, *p* = 0.21), indicating that stress experienced by the subjects did not impact their cage-mates.

We also calculated the SI ratio (a standard evaluation of susceptibility vs. resilience after CSDS; Golden et al., 2011; Peña et al., 2019 to measure the degree to which subjects are socially prone or socially avoidant. Our results revealed a significant main effect of social or isolated housing (Housing effect: F_(1,36)_ = 7.43, *p* = 0.0098; Figure 4B), but no main effect of early-life social defeat (Stress effect: F_(1,36)_ = 0.98, *p* = 0.33). Notably, we also found a trend toward an interaction between whether subjects were exposed to early-life social defeat or not, and whether they were housed in isolation or socially (Housing x Stress: F_(1,36)_ = 3.60, *p* = 0.066), but this interaction fell short of the significant threshold. It was our original expectation that social housing and exposure to chronic social defeat should have compound effects on behavior (specifically stress resilience). Because the interaction closely approached significance, we ran *post hoc* analyses to determine if any particular group contributed more heavily to the main effect of housing, with a specific interest in the role of CSDS+Soc animals. Notably, the CSDS+Soc subjects had significantly higher SI ratios than the Control+Soc subjects (*t*_36_= 2.04, estimate = 1.36, s.e. = 0.67, *p* = 0.049) and CSDS+Isol subjects (*t*_36_= 3.27, estimate = 2.18, s.e. = 0.67, *p* = 0.002). Data are presented following Golden et al. (2011) (Figure 4B), and we draw attention to the high variance in the SI ratio among the CSDS+Soc subjects.

Lastly, the distance traveled and velocity were measured to assess whether social isolation and CSDS impact locomotor activity. We found no main effects of Housing (distance traveled: F_(1,36)_ = 1.02, *p* = 0.32; velocity: F_(1,36)_ = 1.02, *p* = 0.32) or Stress (distance traveled: F_(1,36)_ = 0.26, *p* = 0.61; velocity: F_(1,36)_ = 0.27, *p* = 0.61) and no Stress x Housing interaction (distance traveled: F_(1,36)_ = 0.03, *p* = 0.86; velocity: F_(1,36)_ = 0.03, *p* = 0.86), indicating that neither social isolation nor CSDS influenced locomotion in subjects and cage-mates.

### Effects of social isolation and CSDS on receptor gene expression

After the social approach test, the subjects' brains were assessed for the immediate consequences of social isolation and CDSD on gene expression of the vasopressin, oxytocin, and opioid receptors in the lateral septum. To our surprise, no main effects of Housing, Stress, or Housing x Stress interaction were found for each gene target (Supplementary Table 2). Levels of *avpr1a, oxtr, oprk1, oprm1*, and *oprd1* mRNA in the LS did not significantly differ between groups (Figure 5 and Supplementary Table 2) and did not correlate with SI ratios. There were no significant differences in the gene expression between subjects who exhibited susceptible or resilient phenotypes (Supplementary Figure S1).

**Figure 6:**
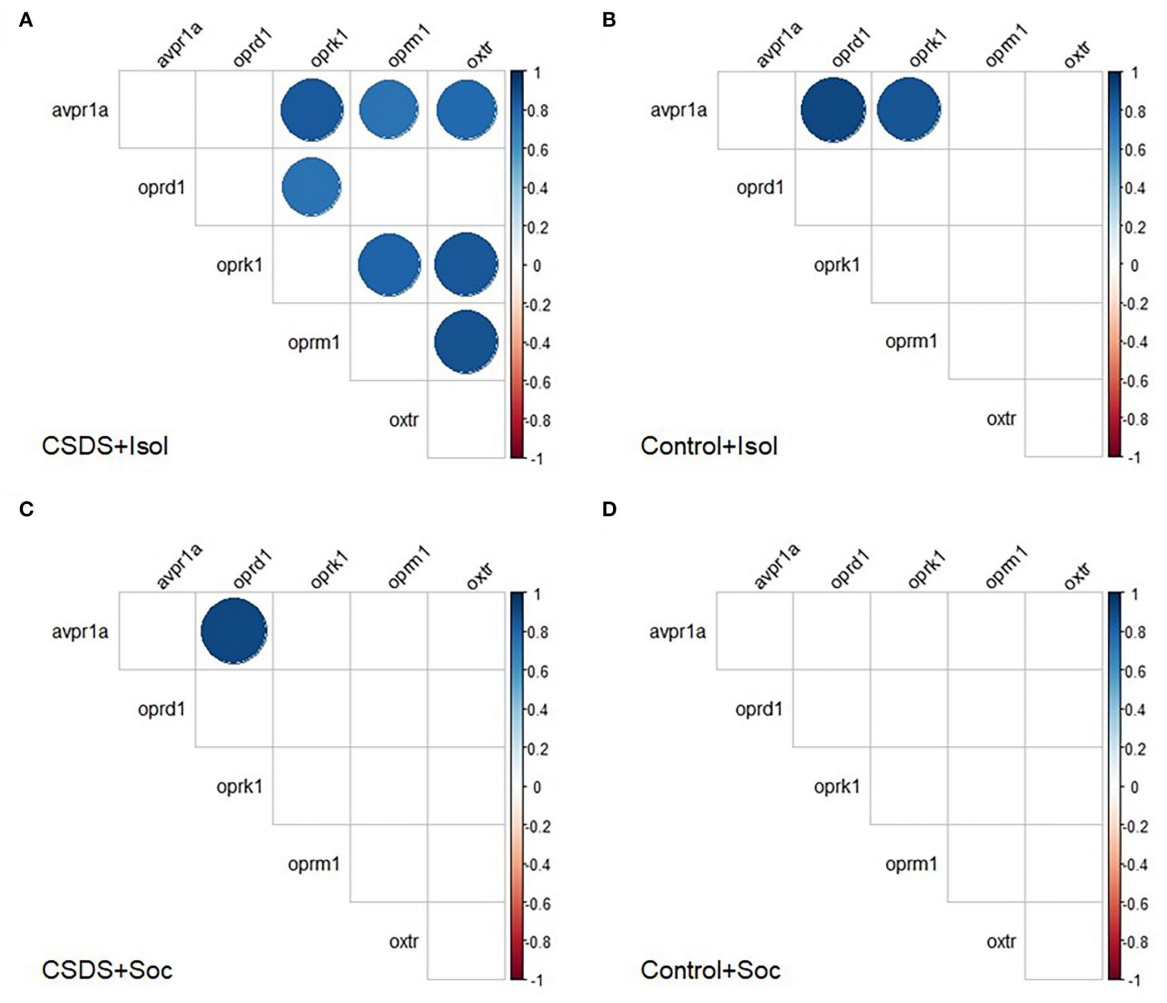
Correlograms separated by group. Pair-wise Pearson correlations with false discovery rate were calculated for mRNA expression and all correlations plotted for the **(A)** CSDS+Isol, **(B)** Control+Isol, **(C)** CSDS+Soc, and **(D)** Control+Soc subjects. Positive correlations are displayed in blue and negative correlations in red color. Color intensity and the size of the circle are proportional to the correlation coefficients. In the right side of each correlogram, the legend color shows the correlation coefficients and the corresponding colors. Statistically non-significant correlations are left blank. The FDR adjusted alpha was α < 0.01 for Control+Isol, α < 0.005 for Control+Soc, α < 0.035 for CSDS+Isol, and α < 0.005 for CSDS-Soc (Benjamini and Hochberg, 1995).

We also investigated the correlations among gene expression within each group to assess the pattern of coordination among these signaling pathways within the LS. Interestingly, several discrete positive correlations between mRNA expression were observed following an FDR adjustment for multiple comparisons (Benjamini and Hochberg, 1995) (Figure 6 and Supplementary Figures S2–S5). Notably, the CSDS+Isol subjects demonstrated broad positive correlations among gene expression across the vasopressin, oxytocin, and opioid receptors, with 7 of the 15 correlations surviving the FDR correction for multiple comparisons. Among these, we highlight the positive correlation between the *oxtr* and *avpr1a* gene expression (r = 0.78, *p* = 0.008; Figure 6A and Supplementary Figure S2), which was not found in animals of the other conditions, suggesting that the combined effect of both isolation and CSDS appears to increase the coordination of gene expression of both nonapeptide receptors within the LS. Furthermore, the CSDS+Isol subjects exhibited significant positive correlations between *oxtr, oprm1*, and *oprk1* (*oxtr* and *oprm1*: r = 0.87, *p* = 0.001, *oxtr* and *oprk1*: r = 0.84, *p* = 0.002, *oprm1* and *oprk1*: r = 0.81, *p* = 0.005; Figure 6A and Supplementary Figure S2). Notably, these relationships were not observed among subjects in the other conditions. Taken together, these data indicate that the combination of CSDS and social isolation uniquely coordinates the gene expression across the vasopressin, oxytocin, and opioid receptor signaling systems within the LS.

In contrast to the CSDS+Isol subjects, few significant correlations were found for gene expression among the subjects assigned to the other groups. However, we did discover a significant positive correlation between *avpr1a* and *oprd1* among the Control+Isol (r = 0.90, *p* = 0.002; Figure 6B and Supplementary Figure S3) and CSDS+Soc (r = 0.91, *p* = 0.0007; Figure 6C and Supplementary Figure S4) animals. Furthermore, both of the socially isolated groups (CSDS+Isol and Control+Isol) showed a significant positive relationship between *avpr1a* and *oprk1* (Control+Isol: r = 0.85, *p* = 0.007, and CSDS+Isol: r = 0.83, *p* = 0.003). Interestingly, our control group (the Control+Soc group) did not show any significant correlations between mRNA expression (Figure 6D and Supplementary Figure S5). These data indicate that social isolation, CSDS, or both impact the coordinated co-expression of one or more of these signaling systems the LS, whereas protection from both stressors leads to a different and uncoordinated pattern of gene expression among them. Moreover, these data indicate that the concordance between oxytocin receptors and the opioid system in the LS increases in parallel among animals suffering from a dual hit of stress, whereas social housing potentially buffers impacts on the relationships between opioid kappa and vasopressin receptors.

## Discussion

In this study, we explored the effects of social isolation, chronic social defeat stress, and their interaction on sociability and neural gene expression. Our results demonstrated that the combination of social isolation overlayed on a background of chronic stress (*via* repeated social defeat) suppressed weight gain, indicating a failure to thrive among animals experiencing this two-hit model of social stress. Moreover, we discovered that subjects exposed to chronic social defeat appear to seek social support. Similarly, although these subjects also appear to develop a pro-social phenotype on average, the variation within these animals indicates that only some have the potential to show this social resiliency, whereas others demonstrate a socially avoidant phenotype. On the other hand, preventing access to social support following a regimen of chronic social stress solely produced social avoidance. No group differences were observed in locomotive behaviors as a response to social isolation and/or CSDS, indicating that both types of stressors specifically affect sociability. Finally, we demonstrated that *avpr1a, oxtr, oprk1, oprm1*, and *oprd1* mRNA levels in the LS did not differ between groups, indicating that social isolation, CSDS, or a combination of both stressors did not elevate or diminish gene expression in any particular group. Within groups, however, social isolation combined with CSDS produced a unique biological signature in which several genes demonstrated significant patterns of co-expression. Considering the high degree of variation in the SI ratio among the CSDS+Soc animals and the notable patterns of gene co-expression, we speculate that differential genetic coordination among these signaling molecules potentially reveals regulatory mechanisms that might modulate the degree to which males are susceptible or resilient to the compounding effects of chronic isolation and social stress.

Suppressed body weight gain has been shown to occur in socially defeated rats and mice, which might be associated with altered food intake, and increased locomotor and exploratory activity (Rygula et al., 2005; Iio et al., 2012; Iñiguez et al., 2014; Alves-dos-Santos et al., 2020). Notably, the DSM-V defines significant weight disorders, specifically weight loss (without dieting) or gain (change of >5% body weight in a month), as a common characteristic of pediatric major depressive disorder (American Psychiatric Association, 2013) suggesting that relatively slow weight gain in developing animals might be evidence of depressive-like behaviors. We showed that prairie vole CSDS+Isol subjects failed to gain body weight between the first and last social defeat sessions. Moreover, we found that social isolation significantly blunted body weight gain in the Control+Isol and CSDS+Isol subjects. Together, these results indicate that social isolation, possibly exacerbated by chronic social stress, contributes to suboptimal weight gain associated with typical development. Locomotor and exploratory activity, including the distance moved and velocity, during the social approach test did not differ as a function of stress exposure between the different group conditions. Thus, the isolation stress-induced decrease of weight gain in our subjects cannot be attributable to the locomotor and exploratory activity. We cannot rule out food intake as a possible factor because we did not include this measure in our analyses. Nevertheless, a failure to thrive is often associated with slow weight gain, delayed development, and behavioral impairments during early development and is attributable to inadequate nutrition, child neglect, and adverse social interactions (Block and Krebs, 2005). Our data suggest that social isolation makes adolescent animals particularly vulnerable to this outcome. More studies are necessary to further examine the degree to which chronic social defeat stress has the potential to heighten social isolation-induced weight gain deficits during adolescence.

Studies that utilize the CSDS paradigm typically maintain subjects co-housed with the resident aggressor for 24 h during sensory stress. Only a handful of these studies on rats and mice have investigated the effects of social support on social defeat-induced social deficits and anxiety-like behaviors (Ruis et al., 1999; de Jong et al., 2005; Nakayasu and Ishii, 2008; Li et al., 2018). However, none evaluated social interactions between the defeated subjects and their cage-mates. We wanted to examine the effects of social support on CSDS-induced sociability and gene expression because the dyadic interactions among cage-mates had the potential to impact both our subjects and their cage-mates, and because identifying if chronic stress can indirectly impact others is important for understanding the scope and span that chronic stress can have on the social environment. Thus, we modified the standardized protocol (Golden et al., 2011) by limiting the sensory stress period to 55 min, returning subjects to either social isolation or pair housing after each social defeat session, and observing social exchanges between the subjects and their cage-mates. Socially housed defeated subjects significantly sought more social support after chronic social defeat stress, when compared to acute social defeat stress and socially housed CSDS-naïve subjects. We interpreted these results to be consistent with consolation-seeking behaviors in stressed animals (Peen et al., 2021). In contrast, cage-mates were unaffected by the treatment of the subjects with whom they were co-housed, indicating that chronic stress impacted the subjects' behavior directly and not the cage-mates indirectly. Taken together, these results highlight the potential benefit of social support as a mechanism that chronically stressed animals use to mitigate the negative consequences of social stressors.

Regardless of the housing condition, subjects from both CSDS+Isol and CSDS+Soc groups took longer to socially approach a novel conspecific. Notably, isolation and CSDS had a compounded effect to promote less resilience and higher rates of susceptible phenotypes, with 80% of CSDS+Isol individuals exhibiting an SI ratio < 0.09. Social housing and CSDS, on the other hand, altered the SI ratio such that CSDS+Soc subjects were on average more resilient (70% exhibiting an SI ratio > 1.1) than the CSDS-Isol subjects (see Figure 4B). Notably, a closer look at the CSDS+Soc group shows profound variation among the SI ratio metric and indicates that despite having social support, some individuals still exhibit a susceptible phenotype (30% exhibiting an SI ratio < 0.9). Thus, social buffering increased the probability that some individuals would demonstrate the resilient phenotype, but others continue to fall victim to the negative consequences of CSDS.

The LS is implicated in several social behaviors and plays a critical role in how animals assess and respond to social contexts (Sheehan et al., 2004). The LS acts as a gating mechanism to control several emotion regulation processes, such as reward, anxiety, sociability, and memory (Sheehan et al., 2004; Rizzi-Wise and Wang, 2021). Among these behaviors, the LS is particularly important for social approach. Higher vasopressin receptor (V1aR) density, but low oxytocin receptor (OTR) density in the LS is correlated with higher levels of social approach in prairie voles (Ophir et al., 2009). Early-life social experience and epigenetic modification of *avpr1a* in the LS are also associated with social approach in prairie voles (Kelly et al., 2020). Recently, we have shown that amplifying neural activity in the LS of prairie voles augments same-sex social approach behaviors [Sailer LL, Park A, Galvez A, Ophir AG (unpublished data)]. In this context, the observation from this study that CSDS causes prairie voles to take longer to approach a novel conspecific regardless of housing condition implicates the LS as a possible site responsible for these results. We, therefore, investigated the gene expression of several signaling systems known to play a key role in regulating LS function and that have been implicated in social approach. To our surprise, we did not find evidence that isolation and/or CSDS impact the overall gene expression of the *avpr1a, oxtr, oprk1, oprm1*, and *oprd1* genes. However, upon closer examination, we found that the gene expression of the *avpr1a, oxtr, oprk1*, and *oprm1* became highly concordant among animals who were isolated and socially defeated. This coordination was not observed for any other treatment group, suggesting that the compounded effects of isolation and CSDS results in an alignment of gene expression across these signaling systems and the potential for co-regulation. It remains to be seen how this coordination of gene expression might impact LS function, but this striking result speaks about the unexpected manner in which social experiences can impact brain organization and function. Furthermore, isolation alone or CSDS alone leads to more limited gene expression concordance, in this case between *avpr1a* and *oprd1*, indicating the presence of related regulatory mechanisms for these gene targets. Also notable, was that socially baseline (Control+Soc) subjects did not show any correspondence or concordance among the gene targets measured in this study. We speculate that the distinct gene expression concordance patterns might represent independent compensatory stress-coping strategies for a single or double hit of stress.

We modified the CSDS paradigm to discriminate between the potentially distinct consequences of social isolation and CSDS, or their combined effects on physical development, comfort-seeking behaviors, stress susceptibility and resilience, and gene expression. We found that the males who remained in social isolation failed to gain a significant amount of body weight across development, and this failure to thrive was exacerbated by simultaneously experiencing CSDS. Although we did not observe a transfer to social stress onto cage-mates or differences in consolation behaviors, we found that socially housed males who experienced CSDS displayed more comfort-seeking behaviors compared to CSDS-naïve socially housed control males. Importantly, socially housed defeated voles presented a split between susceptible and resilient phenotypes. This divergence in social phenotypes was not observed in socially isolated males who experienced CSDS and almost all adopted a susceptible phenotype. Finally, the expression for nearly all the genes we measured was mutually correlated in subjects who experienced both isolation and CSDS, which is a striking contrast to the absence of gene expression correlation in the socially housed CSDS-naïve (i.e., full control) subjects. These data highlight the important impact that isolation or chronic social defeat stress asserts on development independently. Tremendous individual variation for resiliency exists when animals are faced with (social or asocial) stressful life events. These results highlight the importance of understanding why some resilient individuals minimize health-compromised outcomes or mental health disorders, whereas others develop psychopathological disorders after succumbing to stress (Southwick et al., 2016). Social stressors have profound negative consequences on human health and wellbeing, including physical health, anxiety, depression, and post-traumatic stress disorder (Moore et al., 2015). Moreover, the combinatorial impacts of social isolation and chronic social stress could have profound consequences on the development of social behavior, and brain phenotype and function. The COVID-19 pandemic-associated social restrictions have posed serious risks for women and children who are at the most risk to experience escalated domestic violence, and where isolation has prevented victims from reporting abuse and reduced opportunities for seeking support (Sacco et al., 2020; Usher et al., 2020). The short-term effects of such early-life stress are alarming, but it is of great significance to investigate the permanence of these effects and their long-term ramifications.

## Data Availability Statement

The original contributions presented in the study are included in the article/Supplementary materials, further inquiries can be directed to the corresponding authors.

## Ethics Statement

The animal study was reviewed and approved by Animal Care and Use Committee of Cornell University (2013-0102).

## Author contributions

LS and AO conceived, designed the experiments, discussed the results, interpreted the results, and wrote the manuscript. LS conducted behavior testing, brain sectioning, tissue punching, molecular experiments, and data analysis. PP, AP, JM, and AH-L assisted with behavioral scoring and brain sectioning. All authors critically revised the article and approved the final version.

## Funding

This work was supported by funding from the Eunice Kenney Shriver National Institute of Child Health and Human Development to AO (HD079573) and research support to AO from the Cornell University College of Arts and Sciences.

## Conflict of interest

The authors declare that the research was conducted in the absence of any commercial or financial relationships that could be construed as a potential conflict of interest.

## Publisher's note

All claims expressed in this article are solely those of the authors and do not necessarily represent those of their affiliated organizations, or those of the publisher, the editors and the reviewers. Any product that may be evaluated in this article, or claim that may be made by its manufacturer, is not guaranteed or endorsed by the publisher.
