# Supplementary material for: Synergistic consequences of early-life social isolation and chronic stress impact coping and neural mechanisms underlying male prairie vole susceptibility and resilience
Source: Front Behav Neurosci. 2022 Jul 25;16:931549. doi: 10.3389/fnbeh.2022.931549 (PMC9358287; doi:10.3389/fnbeh.2022.931549)
Supplement: Supplementary file 1 [file Data_Sheet_1.DOCX]

**Supplementary Materials: Figures**

**
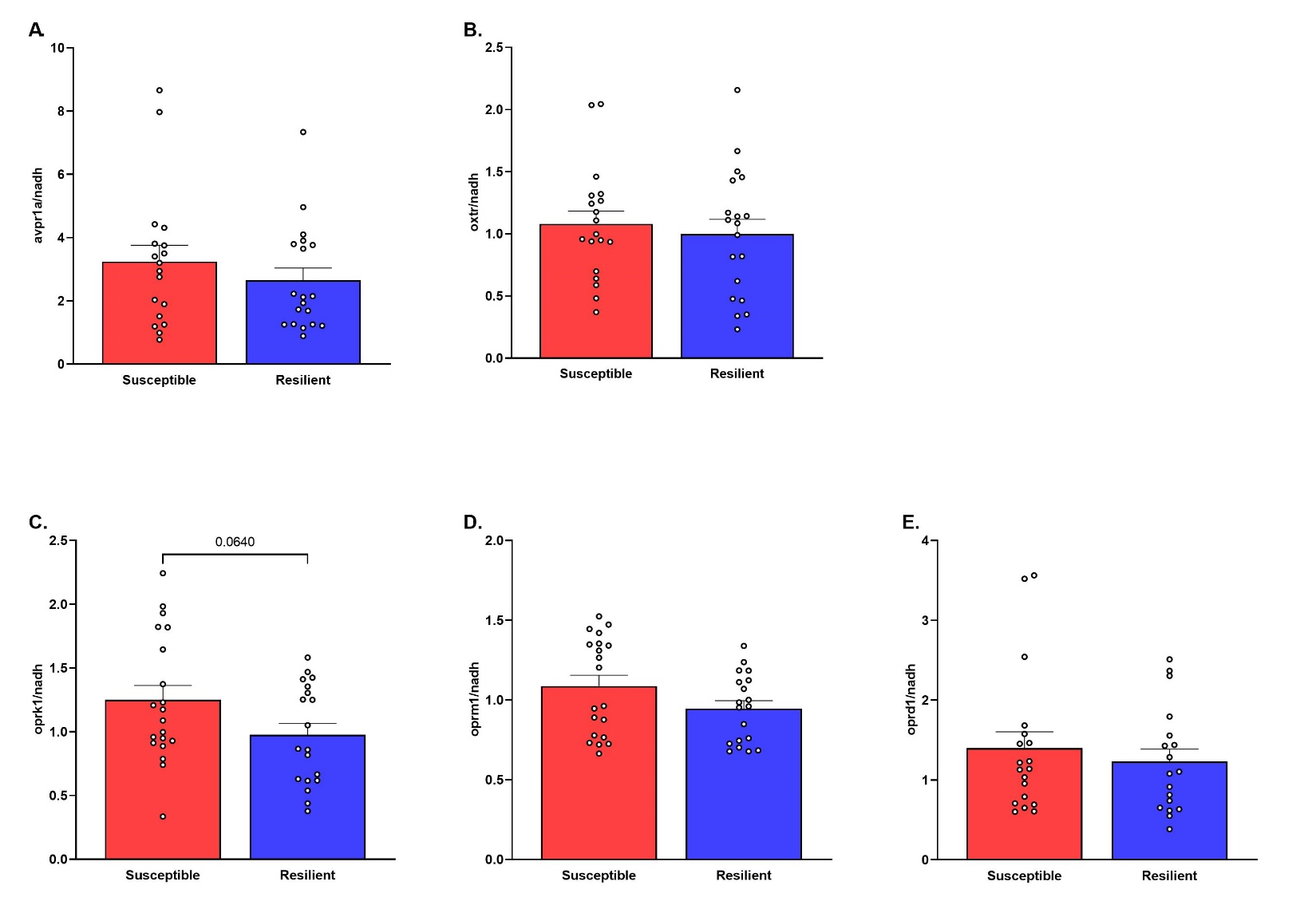
**

**Figure S1 | Gene expression in the lateral septum of susceptible and resilient subjects.** Subjects, regardless of housing (Soc vs Isol) and stress (Control vs CSDS) conditions, were separated by SI ratio scores into susceptible (SI ratio < 0.9) and resilient (SI ratio >1.1) categories to compare mRNA expression of genes measured in Figure 5. Gene expression (mRNA) in the LS did not differ between susceptible and resilient subjects for (A) avpr1a (*t*_35_= 0.93, *p* = 0.36), (B) oxtr (*t*_36_= 0.52, *p* = 0.61), (C) oprk1 (*t*_37_= 1.91, *p* = 0.06), (D) oprm1 (*t*_37_= 1.66, *p* = 0.10), and (E) oprd1 (*t*_35_= 0.64, *p* = 0.52). Data are presented as mean ± SEM and dots represent individual data.

**
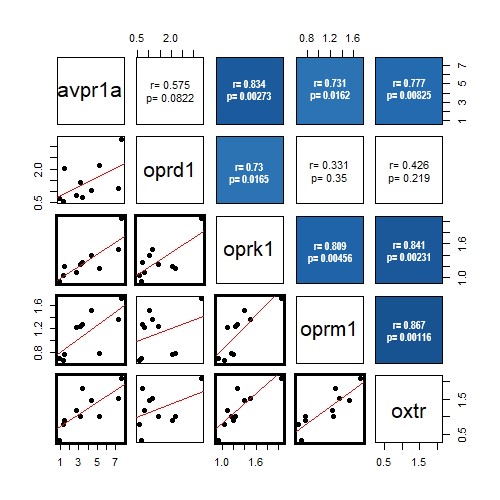
**

**Figure S2** **| Correlation matrix between mRNA gene expression of the CSDS-Isol group.** The upper triangular matrix shows the Pearson correlation coefficients and corresponding *p*-values. The lower triangular matrix is composed of scatter plots with linear regression lines. The FDR adjusted alpha was α < 0.035 for the CSDS+Isol group. Significant relationships correspond to **Figure 6A**, with bold outlines surrounding significant scatterplots, and the blue-colored upper panels representing the strength of the significant relationship.


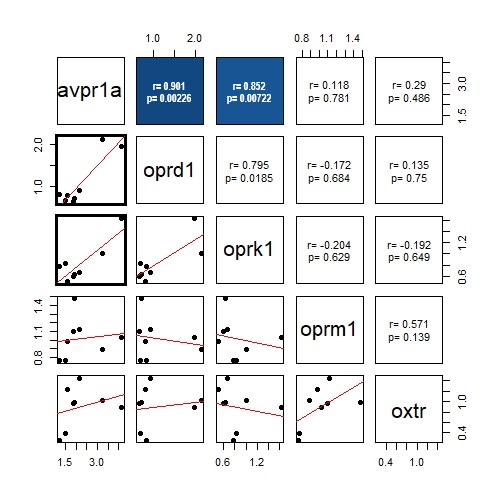


**Figure S3** **|** **Correlation matrix between mRNA gene expression of the Control-Isol group.** The upper triangular matrix shows the Pearson correlation coefficients and corresponding *p*-values. The lower triangular matrix is composed of scatter plots with linear regression lines. The FDR adjusted alpha was α < 0.01 for the Control+Isol group. Significant relationships correspond to **Figure 6B**, with bold outlines surrounding significant scatterplots, and the blue-colored upper panels representing the strength of the significant relationship.


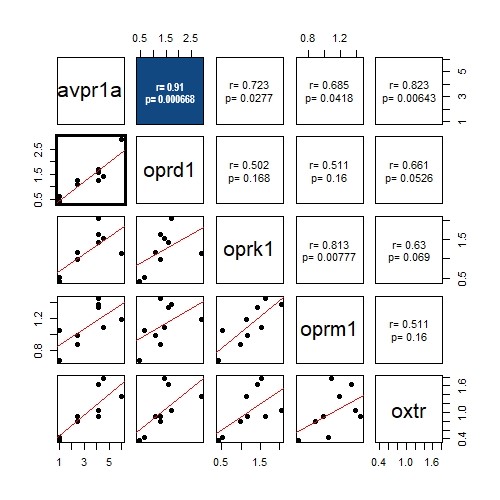


**Figure S4** **|** **Correlation matrix between mRNA gene expression of the CSDS-Soc group.** The upper triangular matrix shows the Pearson correlation coefficients and corresponding *p*-values. The lower triangular matrix is composed of scatter plots with linear regression lines. The FDR adjusted alpha was α < 0.005 for the CSDS-Soc group. Significant relationships correspond to **Figure 6C**, with bold outlines surrounding significant scatterplots, and the blue-colored upper panels representing the strength of the significant relationship.


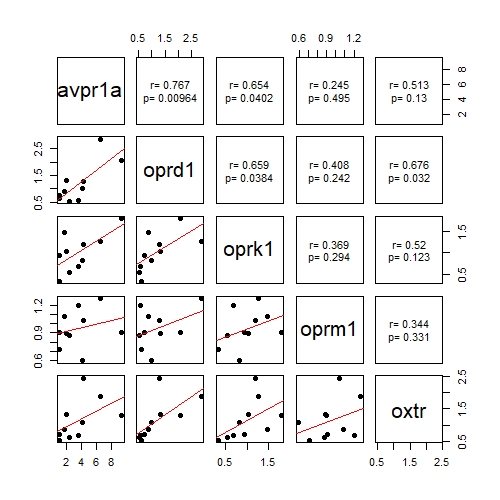


**Figure S5** **|** **Correlation matrix between mRNA gene expression of the Control-Soc group.** The upper triangular matrix shows the Pearson correlation coefficients and corresponding *p*-values. The lower triangular matrix is composed of scatter plots with linear regression lines. The FDR adjusted alpha was α < 0.005 for the Control-Soc group and no gene targets significantly correlated with each other. This figure corresponds to **Figure 6D**.
