# Supplementary material for: Synergistic consequences of early-life social isolation and chronic stress impact coping and neural mechanisms underlying male prairie vole susceptibility and resilience
Source: Front Behav Neurosci. 2022 Jul 25;16:931549. doi: 10.3389/fnbeh.2022.931549 (PMC9358287; doi:10.3389/fnbeh.2022.931549)
Supplement: Supplementary file 2 [file Table_1.DOCX]

**Supplementary Table 1.** Primer sequences used for qPCR.

| **Gene target** | **Sequence** | **Tm** |
| --- | --- | --- |
| *nadh* | **For:** CTATTAATCCCCGCCTGACC | 57.53 |
|  | **Rev:** GGAGCTCGATTTGTTTCTGC | 57.47 |
| *avpr1a* | **For:** GAGGTGAACAATGGCACTAAAACC | 60.56 |
|  | **Rev:** CCAGATGTGGTAGCAGATGAAGC | 61.30 |
| *oxtr* | **For:** TCCAAGGCCAAAATCCGCACGG | 66.16 |
|  | **Rev:** GGCAGAAGCTTCCTTGGGCGC | 66.74 |
| *oprk1* | **For:** AATCGGACAGGAACCGCAG | 60.08 |
|  | **Rev:** ACAGCGGTGATTATGACAGGG | 60.13 |
| *oprm1* | **For:** GGAACACCAGTGACTGTTCTGA | 60.16 |
|  | **Rev:** GATCGGACTGGTTGCCATCT | 59.82 |
| *oprd1* | **For:** ATGTTTGGCATCGTCCGGT | 60.00 |
|  | **Rev:** GTACTTGGCGCTCTGGAAG | 58.24 |
