# Supplementary material for: Synergistic consequences of early-life social isolation and chronic stress impact coping and neural mechanisms underlying male prairie vole susceptibility and resilience
Source: Front Behav Neurosci. 2022 Jul 25;16:931549. doi: 10.3389/fnbeh.2022.931549 (PMC9358287; doi:10.3389/fnbeh.2022.931549)
Supplement: Supplementary file 3 [file Table_2.DOCX]

**Supplementary Table 2.** Statistical outcomes for treatment effects of exposure to chronic social defeat stress and housing (isolated or group housed), and the interaction between them.

|  | **Main effect: Housing** | | **Main effect: Stress** | | **Interaction** | |
| --- | --- | --- | --- | --- | --- | --- |
| **Gene target** | ***F*** | ***p*** | ***F*** | ***p*** | ***F*** | ***p*** |
| *avpr1a* | 0.6629 | 0.421 | 0.2956 | 0.5901 | 2.5739 | 0.1176 |
| *oxtr* | 0.0992 | 0.7546 | 0.0065 | 0.9362 | 2.1856 | 0.1482 |
| *oprk1* | 2.9745 | 0.09317 | 0.0763 | 0.78394 | 0.4544 | 0.50457 |
| *oprm1* | 2.464 | 0.1252 | 0.186 | 0.6689 | 0.0552 | 0.8156 |
| *oprd1* | 0.2864 | 0.596 | 0.0774 | 0.7826 | 0.0277 | 0.8689 |
